# Oleic acid conjugated polymeric photosensitizer for metastatic cancer targeting in photodynamic therapy

**DOI:** 10.1186/s40824-019-0177-7

**Published:** 2020-01-03

**Authors:** Sanghee Lee, Kun Na

**Affiliations:** Department of Biotechnology, Department of Biomedical-Chemical Engineering, Center for Photomedicine, The Catholic University of Korea, 43 Jibong-ro, Wonmi-gu, Bucheon-si, Gyeonggi-do 14662 South Korea

**Keywords:** Photodynamic therapy, Photosensitizer, Cancer therapy, Metastasis cancer

## Abstract

**Background:**

Cancer has been conquered by recent advances in chemotherapy, targeted therapy, and their combinations. However, 90 % of cancer patients die due to cancer recurrence or metastasis. Cancer cell change their metabolic properties to metastasize, changing from conventional glycometabolism to fat metabolism. This is because cancer cells are mainly spread through lymphatic system, which responsible for the absorption and transport of fatty acids and fats. Therefore, cancer cells ahead of metastasis specifically absorb fat to produce energy. Using this property, a photodynamic therapeutic agent conjugated with fatty acids (oleic acid, OA) capable of targeting metastatic cancer cells was developed.

**Main body:**

Polymeric photosensitizer conjugated with OA were composed biocompatible polymers (pullulan) and photosensitizers (chlorin e6, Ce6) (OA-Pullulan-Ce6, OPuC). Pullulan consists of various repeated units, and it is possible to maximize the effects of OA and Ce6 by binding several them to one repeated unit. In this study, the interaction and detection potency of OPuC with cancer cells was confirmed using colon, breast, and lung cancer cell lines. In metastatic cancer cell, OPuC exhibited 3.27-fold higher cellular internalization than non-OA conjugated polymer (Pullulan-Ce6, PuC), however, in negative cell, the variation between OPuC and PuC was negligible despite the existence of OA (1.86-fold). OPuC accumulated in cancer cells could generate singlet oxygen under laser irradiation, resulting in cellular apoptosis and necrosis. Hereby, we proved that OA conjugated polymeric photosensitizer will be a potential metastatic cancer targeting photodynamic therapeutic agent.

**Conclusion:**

Cancer cells actively receive OA conjugated polymeric photosensitizers for fat metabolic pathway, compared with normal cells. Therefore, a new type of polymeric photosensitizer using cancer metabolic properties has potency in metastatic cancer therapy.

## Background

Cancer cell proliferation and invasiveness make it difficult to escape the risk from cancer recurrence or metastasis [1, 2]. Metastatic cancer has a 90 % mortality rate, because it is unclear why cancer spreads and their remedies in clinic [3–5]. Cancer metastasis refers to the dissemination of cancer cells from the primary tumor to other organs via the systemic circulation, specifically a sentinel lymph node [6–8]. Metastasis occurs both early and late stage in primary cancer through the lymphatic pathway, by invading the barriers of nearby blood vessels and lymph node [4, 9]. And the lymph node microenvironment is different condition from the general cancer environment [10, 11].

Lymphatic system is responsible for absorption and transport of fatty acids and fats, so there are abundant lipids [12, 13]. Cancer cells modify metabolic pathway in order to remain alive in lymph node environment. This phenomenon is similar with the characteristics that the metabolic change of cancer to aerobic glycolysis is a well-established marker of cancer [14–16]. To date, it is investigated that metastatic cancer alter their metabolic mechanisms to produce energy by consuming fat to survive in a fatty environment, against other characters that cancer cells generally use glucose as fuel [16, 17]. Taking notice of these cancer cell properties, photosensitizers conjugated fatty acids have been developed, which can be expected to selectively kill metastatic cancer by photodynamic therapy (PDT).

PDT requires a photosensitizer (PS) and a light source with a specific wavelength corresponding to the activity of the PS [18, 19]. PS was accumulated in cellular membrane or intercellular. When intracellular PS was exposed to the specific wavelength, PS chemically react with light and oxygen, and produce a form of reactive oxygen species (ROS), such as singlet oxygen (^1^O_2_) and free radical at disease site. ROS oxidizes cells, causing cellular apoptosis and necrosis. Therefore, polymeric PS was accumulated in cancer cells, and the cells are killed by the generated ROS under laser irradiation. Targeting PSs have cytotoxicity only in PS-accumulated cells and do not damage normal cells without PS [20]. Therefore, cancer targeting PDT can reduce damage in normal tissues and effectively remove tumor as minimally invasive therapy. However, most PSs have some difficulties in clinical use, such as low solubility and low selectivity at disease sites [21].

To overcome these difficulties, conjugating various polymers or targeting moiety have been investigation [22]. Pullulan, a homogenous polysaccharide-based polymer consisting of maltotriose units produced by fungus *Aureobasidium pullulans*, is biodegradable, biocompatible, and soluble in organic solvents. So it has been developed for a drug carrier in the form of nanoparticles [23]. Pullulan has high molecular weight and consists of repeated units. Therefore, various materials can be conjugated at pullulan as a back bone. By conjugating numerous chlorin e6 (Ce6, a kind of PS) and oleic acid (OA, a kind of fatty acid) to large polymer (pullulan), it would be enabled that both PDT effect and targeting ability are more enhanced than single molecules.

In this study, OPuC was developed for inhibition of metastatic cancer proliferation using simple fatty acid. Firstly, we confirmed the physicochemical properties of OPuC and their singlet oxygen species generation effects. Also, we selected cancer cell lines, which have high metastasis risk, and then observed intercellular uptake and interaction with OPuC. Finally, it is demonstrated that OPuC also can generate singlet oxygen in cytoplasm under laser irradiation. Metastatic cancer targeting PDT with OPuC induces cell death, and then their ability applied for all of cancer cells, compared with normal cells.

## Methods

### Materials

Pullulan (molecular weight (MW), 100 kDa) was purchased from Hayashobara (Okayama, Japan). Oleic acid (OA), 1,3-dicyclohexylcarbodiimide (DCC), 4-dimethylaminopyri dine (DMAP), N-hydroxysuccinimide (NHS), dimethyl sulfoxide anhydrous (DMSO), 3-(4,5-dimethyl-2-thiazolyl)-2,5-diphenyl-2H-tetrazolium bromide (MTT) were purchased from Sigma-Aldrich Co. (St. Louis, MO, USA). Chlorin e6 (Ce6) was purchased from Frontier Scientific, Inc., (Salt Lake City, UT, USA). The dialysis membrane (molecular weight cut-off (MWCO), 3.5 kDa) was purchased from Spectrum Laboratories, Inc. (Rancho Dominguez, CA, USA). Singlet Oxygen Sensor Green (SOSG) was purchased from Molecular Probes (Eugene, OR, USA). The ^1^H-NMR spectra were recorded using a Bruker NMR Spectrometer (300 MHz).

### Synthesis and characterization of OPuC

The conjugation of Pullulan-Ce6 (PuC) was performed via DCC/DMAP mediated esterification as previously reported by our group [24]. Briefly, pullulan (100 mg, 21 mM) was completely dissolved in DMSO (10 mL) with DMAP (35.7 mg, 292 μM_ 1.2 fold Ce6 in moles). And then, both Ce6 (69.8 mg, 117 μM) and DCC (29.0 mg, 140 μM_ 1.2 fold Ce6 in moles) are dissolved in DMSO were added to the pre-dissolved pullulan solution and stirred for 48 h at room temperature (RT). After filtration 0.45 μm membrane filtration, the solutions were purified by precipitation in ether (250 mL) three times and then dried in vacuo. To remove remained ether, carried out lyophilization with little water.

Synthesis of OA-Pullulan was preceded to synthesize OPuC. Firstly, pullulan (200 mg, 41 mM), and DMAP (7.1 mg, 6 mM_ 1.2-fold Oa in moles) were dissolved in DMSO (10 mL). OA (165.2 mg, 585 mM) and DCC (144.8 mg, 700 μM_ 1.2-fold OA in moles) were dissolved in DMSO (1 mL). Both the pullulan and OA solutions were mixed together, followed by stirring for 48 h at RT. After reaction, the solution was purified by precipitation and vacuo. To conjugate Ce6 with OA-Pullulan, OA-Pullulan (100 mg), DMAP (1.3 mg, 1 mM), Ce6 (64.8 mg, 11 mM), and DCC (26.9 mg, 13 mM) were completely dissolved in DMSO 10 mL. After 48 h reaction time, the final solution was purified in the same way as the described method of purifying PuC. The synthesis of PuC and OPuC were evaluated by recording the ^1^H-NMR spectra through a Bruker NMR Spectrometer (Bruker, Germany). Zeta potential of OPuC was measured using dynamic light scattering (DLS, Zetasizer Nano ZS (Malvern Instruments, Malvern, UK) in DI water.

### Singlet oxygen generation efficacy of OPuC

To measure the singlet oxygen generation efficacy of OPuC, singlet oxygen sensor green (SOSG) solution (10 μM) was mixed with 1 mL of free Ce6 and OPuC at 10 μg mL^− 1^ of Ce6 equivalent. The absorbance of samples was analyzed by UV-vis spectroscopy (UV-2450, Shimadzu, Japan) to quantify of Ce6 concentrations at 664 nm wavelength. Each sample was irradiated with 20 mW cm^− 2^ of a 670 nm laser source (Fiber Coupled Laser Modules, LaserLab®, Seoul, Korea) for 200 s. The fluorescence intensity of SOSG (λex = 504 nm, λem = 525 nm) was detected using fluorescence spectroscopy (RF-5301, Shimadzu, Japan).

### Cell culture and incubation conditions

Fetal bovine serum (FBS), antibiotics (penicillin/ streptomycin), and Dulbecco’s phosphate buffered saline (DPBS) were purchased from Gibco BRL (Invitrogen Corp., Carlsbad, CA, USA). L929 cells (mouse connective tissue normal cell line, KCLB no.10001), A549 (human lung carcinoma cell line, KCLB no.10185), PANC-1 (human pancreas carcinoma cell lines, KCLB no.21469), and HCT116 (human colon carcinoma cell line, KCLB no.10247) were obtained from the Korean Cell Line Bank (KCLB). L929, PANC-1 cells were cultured in DMEM (Dulbecco Modified Eagle Medium) and A549, HCT116 cells were cultured in RPMI 1640 (Roswell Park memorial Institute 1640 Medium) supplemented with 10% FBS and 1% penicillin/ streptomycin. Cells were cultured at 37 °C in 5% CO_2_ and changed fresh medium every 2 to 3 days. PuC and OPuC were dissolved in DMSO and diluted in serum-free (SF) medium until the DMSO concentration reached under 0.1%. All reported concentrations referred to free Ce6 equivalents. Untreated cells were kept in the dark and used as a reference standard.

### In vitro cellular uptake of OPuC

To observe the cellular uptake of OPuC, flow cytometry and confocal laser scanning microscope (CLSM) were carried out. Different type of cells (1.0 × 10^5^ cells/well in a 12-well plates) were incubated with PuC or OPuC (Ce6 conc. 5 μg mL^− 1^) for 4 h at 37 °C. Cells were washed three times, harvested with DPBS, and transferred to FACS tubes. All samples were analyzed by a Becton-Dickinson FACS CantoII (San Jose, CA, U.S.A). For each sample, 10,000 cells (gated events) were counted, and Ce6 fluorescence was detected with logarithmic settings (APC (Ce6), λem = 675 nm). Each experiment was analyzed statistically using FACS Diva software (BD).

L929 and HCT116 cells were grown at a density of 1.0 × 10^5^ cells/well on 25 mm sterile round shape cover glasses inserted in a 6 well plate at 37 °C for 18 h. SF-media containing PuC or OPuC (equivalent to Ce6 conc. 5 μg mL^− 1^) was added, and cells were incubated for 4 h. After the incubation medium was removed, cells were washed with DPBS, fixed for 10 min with 4% paraformaldehyde solution at room temperature, and stained with 4,6-diamidino-2-phenylindole (DAPI 1 μL, 3.63 mM) for 2 min. After washing, the cover glasses were placed onto slides for imaging with a confocal laser scanning microscope (CLSM, LSM 710 Meta, Carl Zeiss, Germany). Fluorescent images were analyze using the LSM Image Browser software (Carl Zeiss, Germany).

### In vitro phototoxicity of OPC

A549, PANC-1, HCT116, and L929 cells (2 × 10^4^ cells/well in 48-well plates) were incubated with PC or OPuC (0.10, 0.25, 0.50, 0.75, 1.00, 1.50, or 2.00 μg mL^− 1^ of Ce6) for 4 h at 37 °C. After the incubation, the media were replaced with a fresh culture medium. Cells were irradiated with a 670 nm laser source (1 J cm^− 2^) and then incubated for 24 h. The MTT solution (1 mg mL^− 1^) was added to each well and incubation for 3 h, followed by replacement with DMSO. The absorbance of the MTT dye at 570 nm was measured using a microplate reader (Bio-Tek, VT, USA) to determine cell viability.

### Statistical analysis

Experimental Data are presented as mean ± standard error of the mean for results obtained from three independent trials unless otherwise indicated. The statistical significance was determined using one-way analysis with *p*-values < 0.05 as the level of significance. (∗*p* < 0.05, ∗∗*p* < 0.01, ∗∗∗*p* < 0.001).

## Results

### Synthesis and characterization of OPuC

**Fig. 1:**
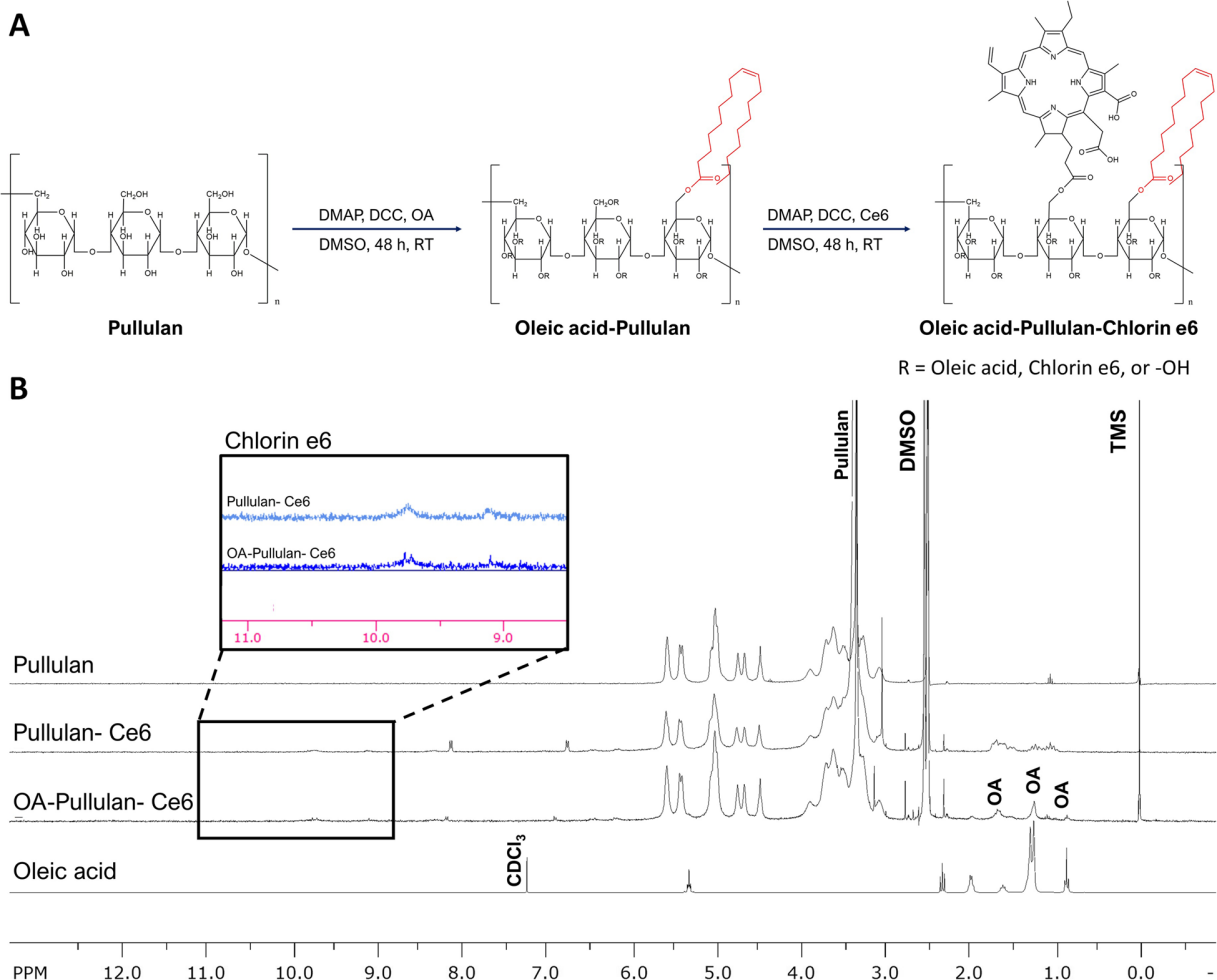
Characterization of Oleic Acid-Pullulan-Ce6. (**a**) Synthetic route of OPuC. OA is firstly conjugated with Pullulan and then Ce6 is conjugated with the remaining hydroxyl group. (**b**) ^1^H-NMR analysis of OPuC and PuC in DMSO-d_6_, and Oleic acid in CDCl

**Fig. 2:**
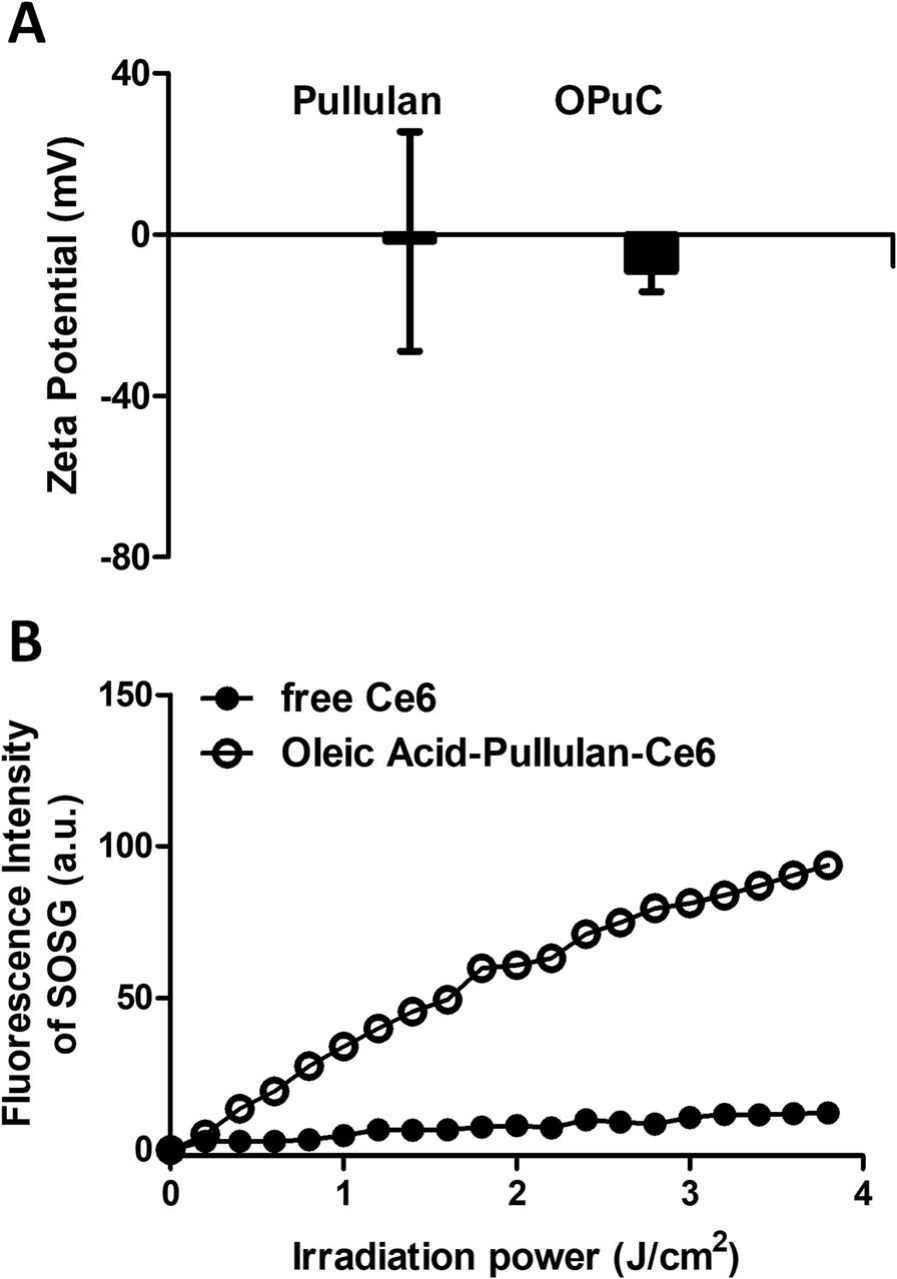
Characterization of OPuC (**a**) Zeta potential of pullulan and OPuC at 1 mg mL^− 1^ (**b**) Singlet oxygen generation (^1^O_2_) measurement of free Ce6, and OPuC at 10 μ mL^− 1^ of Ce6 by mixing with singlet oxygen sensor green (SOSG, conc; 10 μM) in deionized water (DI water). All light irradiations were performed at 670 nm. (4 J cm^− 2^, 20 mW cm^− 2^, 200 s)

The synthesized OPuC was analyzed by ^1^H-NMR and quantified the concentration of Ce6 via UV-vis spectrometer. Peak of ^1^H-NMR showed conjugated Ce6 on PuC and OPuC, and then the OPuC spectra contained the peak of OA (Fig. 1b). Despite pullulan had neutral net charge, OPuC had slightly anion charge by conjugating OA and Ce6, containing carboxyl groups (Fig. 2a). Further, ROS generation efficiency of the OPuC was confirmed in aqueous condition using the singlet oxygen sensor green (SOSG). Fluorescence intensity of SOSG on free Ce6 did not show meaningful increase, however, OPuC effectively produced ROS depending on laser irradiation time (Fig. 2b). This is because, free Ce6 did not dissolve but rather aggregated in aqueous condition, occurring quenching fluorescence intensity due to π-π interactions. However, the OPuC enhance their solubility in water, so that successfully generated ROS.

### Interaction of OPuC with cancer cells

**Fig. 3:**
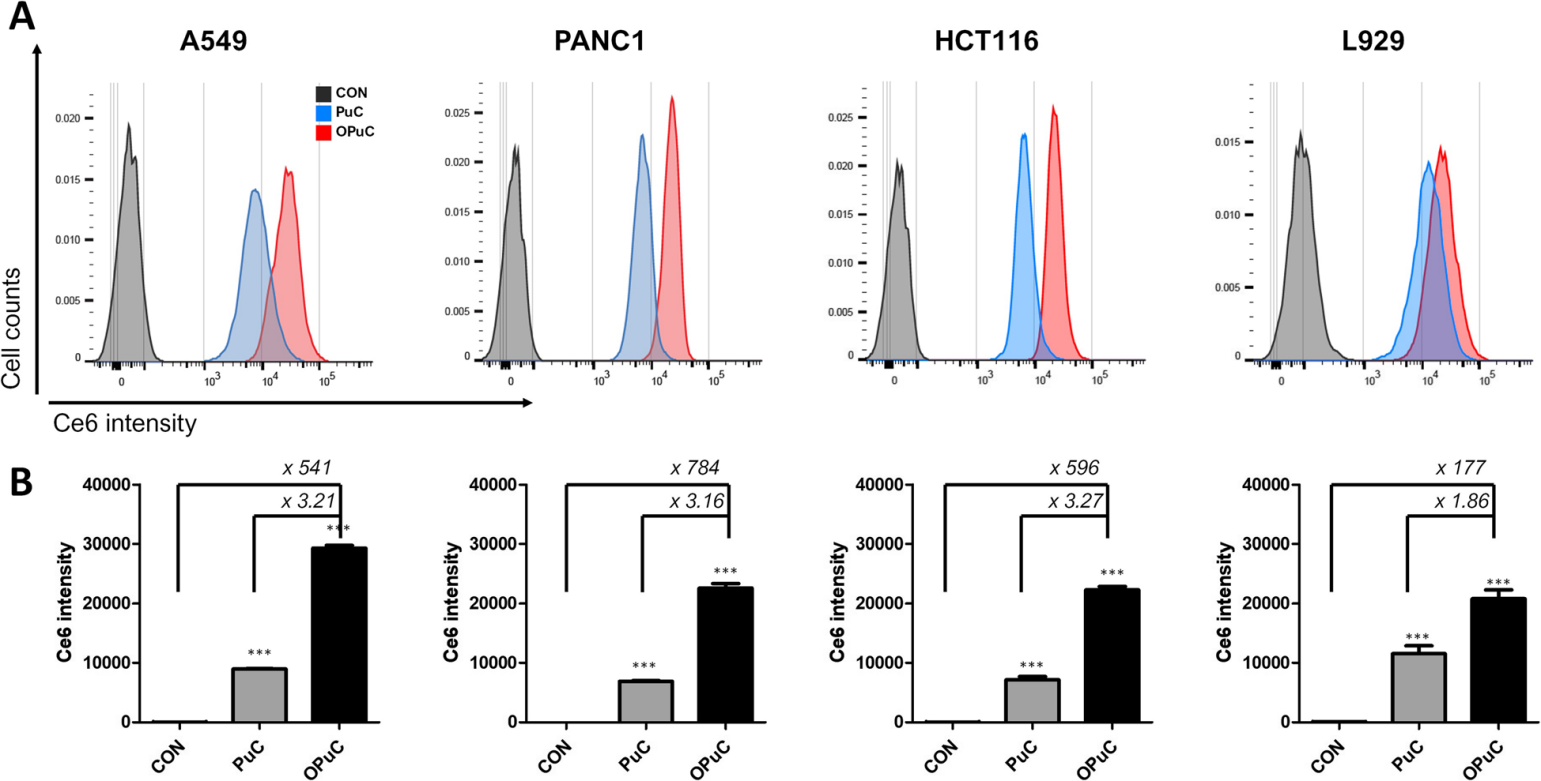
Cellular uptake of OPuC in A549, PANC-1, HCT116 (positive cancer cell lines), and L929 (negative cell lines). Flow cytometry analysis after treatment of PuC or OPuC at 5 μg mL^− 1^ of the Ce6 concentration for 4 h. (**a**) Representative histogram for the Ce6 fluorescence intensity in cytoplasm. (**b**) Bar graph presents the mean of Ce6 intensity and quantifies increase rate

Cancer cells prepare metastasis to other organs by modifying their metabolic properties, consuming fatty acid as fuel. To determine the affinity between OA and cancer cells, cell internalization of OPuC was analyzed by flow cytometry in cancer cell lines that are known to be well metastasized, such as lung (A549), pancreas (PANC-1), colon cancer (HCT116) (Fig. 3b). And the fluorescence intensity of Ce6 in cytoplasm was quantified and presented in a bar graph (Fig. 3b). Overall, the Ce6 fluorescence intensity in OPuC-treated cancer cells was significantly increased, compared with PuC-treated cancer cells. However, there was no meaningful difference in L929 (normal cell, mouse fibroblast) that were incubated with PuC or OPuC. The fluorescence intensity of OPuC-treated HCT116 cells raised around 3.27-fold more than PuC-treated, but L929 cells increased 1.86-fold, which means L929 had non-specific interaction with OA by hydrophobic interaction.

**Fig. 4:**
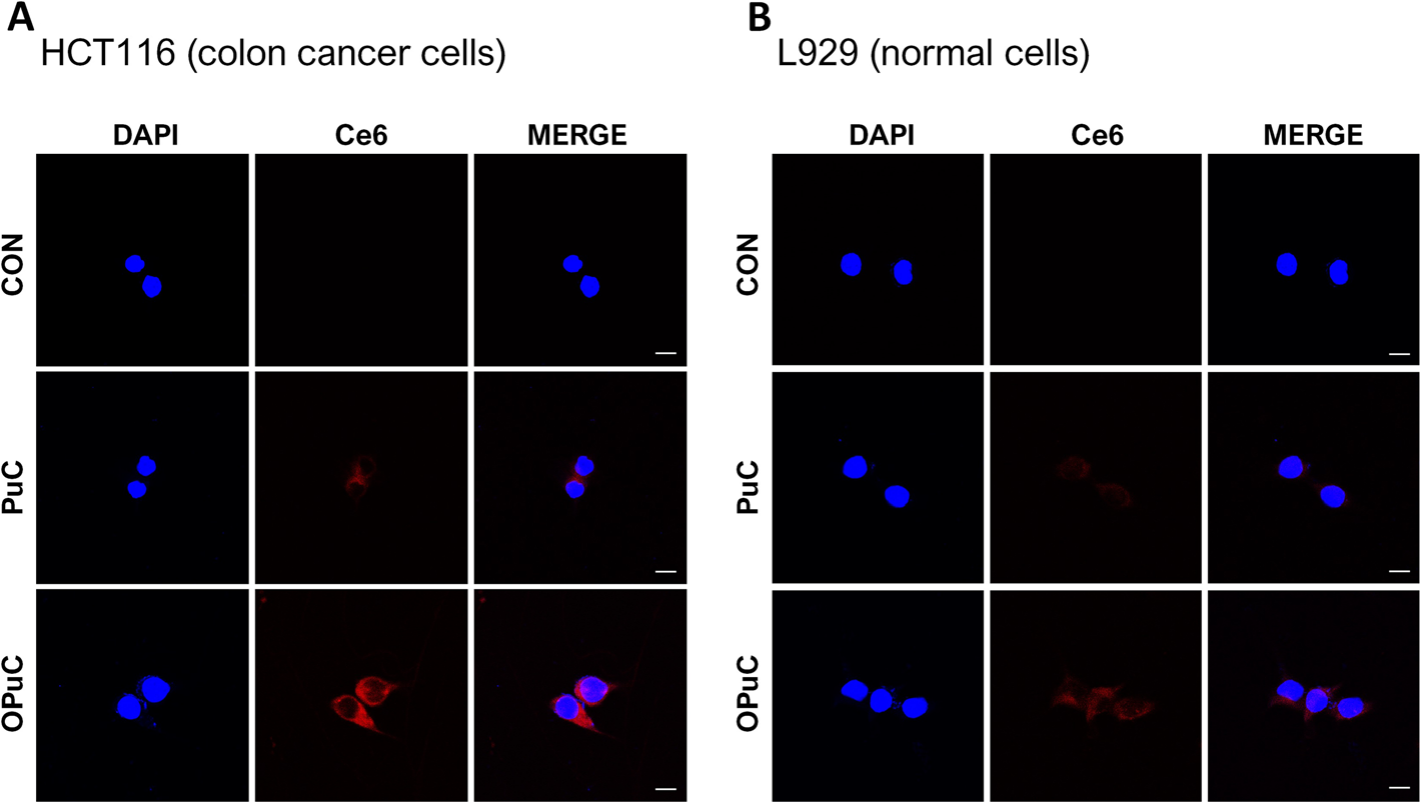
Cellular uptake of OPuC by Confocal Laser Scanning Microscopy images in HCT116 (**a**, positive cell line) and L929 (**b**, negative cell line) after treatment PuC or OPuC (5 μg mL^− 1^ of Ce6) for 4 h. Nucleus were stained DAPI (blue) and accumulated intracellular PuC or OPuC were brighten red. Merge images present the interaction with cells. The scale bar is 10 μm

To visualize the targeting ability of OPuC, both HCT116 cells (positive cell lines, Fig. 4a) and L929 (negative cell lines, Fig. 4b) were incubated with PuC or OPuC and observed via CLSM. Like flow cytometry data, OPuC was efficiently absorbed into cells than PuC. Interestingly, images of HCT116 cells treated OPuC significantly brighter than those of L929 cells, but PuC images had no meaningful difference in two cells.

### Intracellular ROS generation of OPuC

**Fig. 5:**
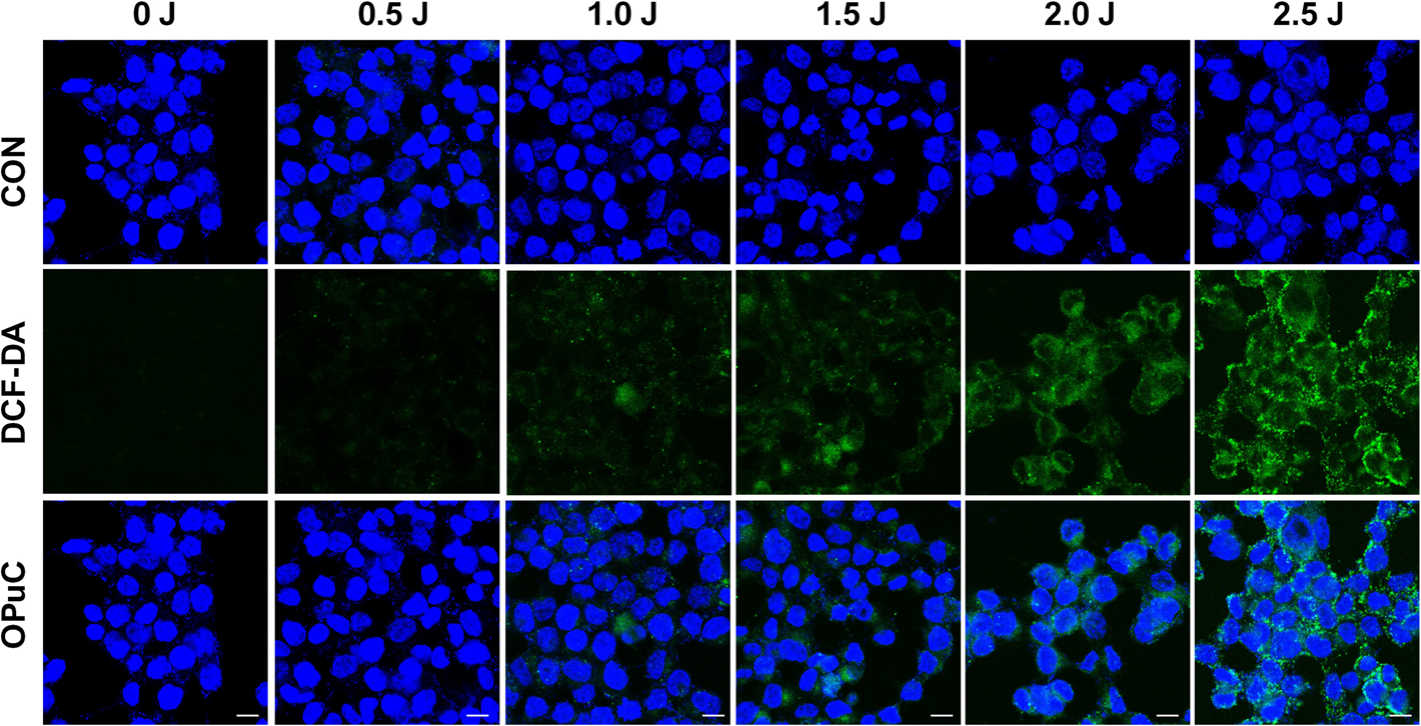
Intracellular ROS generation of OPuC. Fluorescence microscopic images of HCT116 cells after treatment OPuC (2 μg mL^− 1^ of Ce6) for 4 h to detect ROS type_1_ (DCF-DA, conc 2.5 μM) depending on increase of laser powers. The scale bar is 10 μm

OPuC can be generated ROS intracellular condition to induce cell death. DCF-DA penetrated live cell membrane is oxidized and emits luminous green fluorescence in the presence of non-specific ROS. So, we confirmed ROS generation via DCF-DA, and determined a potency of PDT (Fig. 5). In case of HCT116 cells, cells fully incubated with the same concentration with OPuC and irradiated laser at various laser power, emitting strong green fluorescence. The fluorescence intensity was gradually increased depending on laser power. The strong laser intensity allows the PSs to better form ROS by chemical reactions of light and oxygen, which directly related to cell death.

### Phototoxicity of OPuC reflects the targeted therapy

**Fig. 6:**
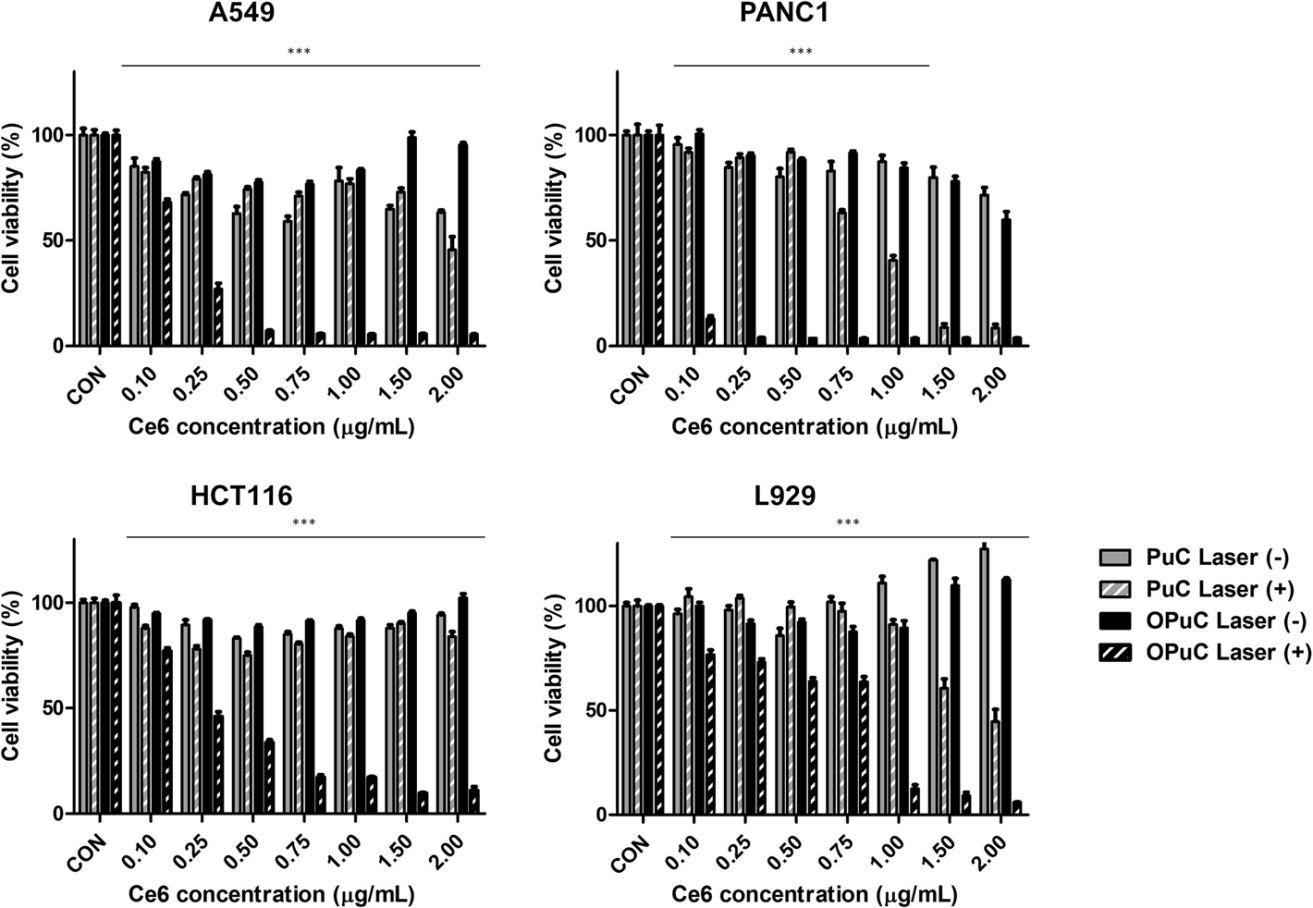
In vitro cancer cell specific phototoxicity of OPuC. MTT assay at various concentration of Ce6 under laser irradiation of 1 J cm^− 2^ (10 mW cm^− 2^, 100 s) in A549 (lung cancer), PANC-1 (pancreatic cancer), HCT116 (colon cancer), and L929 (normal fibroblast

With confirming cancer cell specific uptake of OPuC and singlet oxygen generation, we finally demonstrated the relation of cancer cell death using MTT assay (Fig. 6). Cancer cell death was caused by PDT upon laser irradiation in the specific wavelength range. So, we incubated cells with PuC or OPuC, subsequently irradiating laser at 670 nm. As shown Fig. 6, PuC and OPuC without laser groups did not induce cell death in all cell lines, however, cell viability was dramatically decrease in both PuC and OPuC with laser groups. Focusing on OPuC with laser in cancer cell lines (A549, PANC-1, and HCT116), cell death indicated at 0.1 μg mL^− 1^ Ce6 concentration of OPuC, and then cell viability gradually decreased depending on the increase of Ce6 concentration. However, in L929 cells, it was confirmed that cell viability was maintained up to 0.75 μg mL^− 1^ Ce6 concentration of OPuC. Based on the analysis of flow cytometry and confocal images, cancer cell absorbed more OPuC under the same conditions, which showed stronger phototoxicity under irradiated same power laser. However, normal cells showed low sensitivity to phototoxicity of OPuC.

## Discussion

Cancer cell was changed every condition to rapidly proliferate. Metastatic cancer modifies their metabolic mechanism to survive in lymphatic system. So, we kept an eye on this cancer’s properties, developing metastatic cancer targeting PDT agents. Fat conjugated PS (OA-Pullulan-Ce6, OPuC) were developed using the properties of metastatic cancers that ingested fat. Free Ce6 have low solubility in water and low specificity at disease sites. However, Pullulan enhanced the solubility of PS, decreasing the fluorescence quenching effects. Thus, OPuC fulfill the PS’s ability in body fluidic conditions, so it can be utilized as a therapeutic agent for targeted PDT.

The synthesized OPuC was specifically accumulated in cancer cells, and successfully generated effective ROS. Intracellular ROS was enhanced in the laser dependent manners, and eventually induced cell death. In the phototoxicity of OPuC with laser at 0.50 μg mL^− 1^ of Ce6, the survival rate was 7.07% in A549, 3.61% in PANC-1, and 33.48% in HCT116 (cancer cell, positive cell). But the survival rate was 64.0% in L929 (normal cell, negative cell) at the same concentration of Ce6. We can conclude that OPuC has specific interactions with cancer cells originated from other organs. Thus, OPuC deserve much consideration on the applicability of metastatic cancer PDT.

## Conclusions

In previous studies, the special characters of metabolic pathway in metastatic cancer provided clues that targeted metastatic cancer therapy may be possible. We applicated this discovery in PDT and have developed a method to specifically kill metastatic cancer cells. We designed OPuC to target metastatic cancer using their metabolic properties consuming fatty acids. A variety of cancer cells were effectively detected and interacted with OPuC due to fatty acid (i.e., Oleic acid). Further, low concentration of OPuC successfully caused cancer cell death through ROS generation upon laser in vitro. In order to utilize our study in clinic, therapeutic effects will be maximized when the premise that cancer cell have metastatic properties. Moreover, they must precede that OPuC is accumulated into cancer cells in cancer patient as well as it is effective enough to prevent metastasis. However, an approach of cancer therapy using metabolic feature is quite meaningful.

## Abbreviations

DCF-DA: 2`, 7`–Dichlorofluorescein diacetate
OPuC: Oleic acid-Pullulan-Ce6
PDT: Photodynamic therapy
PS: Photosensitizer
PuC: Pullulan-Ce6
ROS: Reactive oxygen species
SOSG: Singlet oxygen sensor green
